# Sarcopenia in Autoimmune and Rheumatic Diseases: A Comprehensive Review

**DOI:** 10.3390/ijms21165678

**Published:** 2020-08-07

**Authors:** Hyo Jin An, Kalthoum Tizaoui, Salvatore Terrazzino, Sarah Cargnin, Keum Hwa Lee, Seoung Wan Nam, Jae Seok Kim, Jae Won Yang, Jun Young Lee, Lee Smith, Ai Koyanagi, Louis Jacob, Han Li, Jae Il Shin, Andreas Kronbichler

**Affiliations:** 1 Yonsei University College of Medicine, Seoul 03722, Korea; hjj622@yonsei.ac.kr; 2 Laboratory Microorganismes and Active Biomolecules, Sciences Faculty of Tunis, University Tunis El Manar, Tunis 2092, Tunisia; kalttizaoui@gmail.com; 3 Department of Pharmaceutical Sciences and Interdepartmental Research Center of Pharmacogenetics and Pharmacogenomics (CRIFF), University of Piemonte Orientale, 28100 Novara, Italy; salvatore.terrazzino@uniupo.it (S.T.); sarah.cargnin@uniupo.it (S.C.); 4 Department of Pediatrics, Yonsei University College of Medicine, Seoul 03722, Korea; AZSAGM@yuhs.ac; 5 Department of Rheumatology, Wonju Severance Christian Hospital, Yonsei University Wonju College of Medicine, Wonju 26426, Korea; namsw@yonsei.ac.kr; 6 Department of Nephrology, Yonsei University Wonju College of Medicine, Wonju 26426, Korea; ripplesong@yonsei.ac.kr (J.S.K.); kidney74@yonsei.ac.kr (J.W.Y.); junyoung07@yonsei.ac.kr (J.Y.L.); 7 The Cambridge Centre for Sport and Exercise Science, Anglia Ruskin University, Cambridge CB1 1PT, UK; Lee.Smith@anglia.ac.uk; 8 Research and Development Unit, Parc Sanitari Sant Joan de Déu, CIBERSAM, 08830 Barcelona, Spain; a.koyanagi@pssjd.org (A.K.); louis.jacob.contacts@gmail.com (L.J.); 9 ICREA, Pg. Lluis Companys 23, 08010 Barcelona, Spain; 10 Faculty of Medicine, University of Versailles Saint-Quentin-en-Yvelines, 78000 Versailles, France; 11 University of Florida College of Medicine, Gainesville, FL 32610, USA; lih2@ufl.edu; 12 Department of Internal Medicine IV (Nephrology and Hypertension), Medical University Innsbruck, 6020 Innsbruck, Austria; Andreas.Kronbichler@i-med.ac.at

**Keywords:** sarcopenia, rheumatic disease, autoimmune disease, rheumatoid arthritis, inflammatory bowel disease, type 1 diabetes

## Abstract

Sarcopenia refers to a decrease in skeletal muscle mass and function. Because sarcopenia affects mortality, and causes significant disability, the clinical importance of sarcopenia is emerging. At first, sarcopenia was recognized as an age-related disease but, recently, it has been reported to be prevalent also in younger patients with autoimmune diseases. Specifically, the association of sarcopenia and autoimmune diseases such as rheumatoid arthritis has been studied in detail. Although the pathogenesis of sarcopenia in autoimmune diseases has not been elucidated, chronic inflammation is believed to contribute to sarcopenia, and moreover the pathogenesis seems to be different depending on the respective underlying disease. The definition of sarcopenia differs among studies, which limits direct comparisons. Therefore, in this review, we cover various definitions of sarcopenia used in previous studies and highlight the prevalence of sarcopenia in diverse autoimmune diseases including rheumatoid arthritis, spondyloarthritis, systemic sclerosis, inflammatory bowel disease, and autoimmune diabetes. In addition, we cover the pathogenesis and treatment of sarcopenia in autoimmune and rheumatic diseases. This review provides a comprehensive understanding of sarcopenia in various autoimmune diseases and highlights the need for a consistent definition of sarcopenia.

## 1. Introduction

The loss of muscle mass and function with aging is a natural phenomenon. In the seventh and eighth decade of life, muscle strength decreases by 20–40% and the degree of reduction increases gradually [1]. The decrease in skeletal muscle mass, strength, and function associated with aging is termed sarcopenia [2,3,4]. Sarcopenia is associated with daily life disability, falls in older people, and a high risk of all-cause mortality [5,6]. Furthermore, there is a financial burden associated with sarcopenia because the hospitalization costs for patients with sarcopenia are significantly higher than those without sarcopenia [7]. As the clinical importance of sarcopenia has become apparent, it is now considered to be a disease entity in the International Classification of Diseases (ICD) [8]. Although sarcopenia is usually considered to be an age-related disorder, younger people with various clinical conditions also suffer from sarcopenia. Age-related sarcopenia with no other causes is called ”primary sarcopenia”, whereas when one or more other causes such as malnutrition are evident, sarcopenia is classified as ”secondary sarcopenia” [4]. In many cases, sarcopenia is age-related and is also a multifactorial problem [9]. It is well known that endocrine diseases or malignancies promote sarcopenia [10]. Likewise, chronic inflammation is also a paramount risk factor for sarcopenia [10,11]. From this point of view, autoimmune diseases with persistent chronic inflammation due to autoreactive immune response, could be a risk factor for sarcopenia. Indeed, a recent study showed that having any autoimmune disease was associated with sarcopenia with an odds ratio (OR) of 1.83 [12]. In addition, the association between rheumatoid arthritis (RA) and sarcopenia is well established. Nevertheless, to date, there are no comprehensive reviews regarding the relationship of sarcopenia and autoimmune diseases. This review addresses this gap and covers the association between sarcopenia and autoimmune or rheumatic diseases. This review mainly addresses RA rather than other diseases due to a difference in the sufficiency of studies.

## 2. Definition and Diagnosis of Sarcopenia

In 1989, Irwin Rosenberg first coined the term “sarcopenia” (Greek “sarx” or flesh and “penia” or loss) to define the decrease of skeletal muscle mass, however, until now there has been no unified definition or diagnosis of sarcopenia [2,3]. Baumgartner et al. defined sarcopenia based on skeletal muscle mass [13]. The skeletal muscle mass index (SMI) was defined as appendicular skeletal muscle mass (ASM)/height^2^ (kg/m^2^), and sarcopenia was defined if the SMI was two standard deviations below the mean of a gender-specific reference group [13]. After a few years, Janssen et al. proposed cut points of height-adjusted skeletal muscle mass that were associated with a physical disability risk [14]. Later, several consensus groups proposed a definition using both muscle mass and function [4,15,16,17,18]. In 2010, the European Working Group on Sarcopenia in Older People (EWGSOP) defined sarcopenia as “a syndrome characterized by progressive and generalized loss of skeletal muscle mass and strength with a risk of adverse outcomes such as physical disability, poor quality of life and death”, a definition that now represents the most widely used definition in the clinical realm [4]. According to the EWGSOP, the definition of sarcopenia was (1) having low muscle mass and (2) having low muscle strength or low physical performance [4]. The Foundation of the National Institute of Health (FNIH) [15], the International Working Group on Sarcopenia (IWGS) [16], and the European Society on Clinician Nutrition and Metabolism special interest groups (ESPEN SIG) [17] also proposed a definition for sarcopenia that contained both muscle mass and function. The Asian Working Group for Sarcopenia (AWGS) took a similar approach for sarcopenia but proposed a new and more appropriate cut-off value for Asians, considering that already proposed cut-points had been calculated from Caucasian data [18]. Recently, the European Working Group on Sarcopenia in Older People 2 (EWGSOP2) revised the definition of sarcopenia and characterized it by (1) low muscle strength and (2) low muscle quantity or quality [19]. This change reflected the study results that muscle strength is a more important prognostic factor than muscle mass [20,21,22,23].

## 3. Epidemiology of Sarcopenia in Autoimmune and Rheumatic Disease Patients

Table 1 shows the prevalence of sarcopenia in autoimmune and rheumatic diseases. The prevalence of sarcopenia varies based on the type of autoimmune disease, and also the different definitions used, as well as the subject groups analyzed. Overall, the clinical definitions are heterogeneous. On the one hand, many studies have defined sarcopenia using only one aspect, muscle mass or lean mass which are calculated using the SMI or the free fat mass index (FFMI), respectively. On the other hand, other studies have defined it using muscle mass plus muscle strength (e.g., handgrip strength) and performance (e.g., TUG, timed up and go). Furthermore, the cut-off value for sarcopenia differs among studies. The column ”definition of sarcopenia” highlights the respective criteria used to define sarcopenia. Krajewska-Włodarczyk et al. demonstrated that the difference in definition affected study results [24]. In female patients with psoriatic arthritis (PsA), the prevalence of sarcopenia was 13.7, 49.0, and 43.1%, respectively, when it was diagnosed using the following different definitions of SMI: (1) appendicular muscle mass/height^2^ < 5.45 kg/m^2^ [13], (2) skeletal muscle mass/weight × 100 < 27.6% [25], (3) skeletal muscle mass/weight × 100 < 27.6% with TUG > 14 s [25]. The heterogeneity of prevalence due to the different definitions of sarcopenia makes the need for a unified definition and diagnostic criteria for sarcopenia urgent.

### 3.1. Rheumatoid Arthritis

Among the studies investigating the prevalence of sarcopenia in autoimmune diseases, most studies have been performed in RA patients. Dao et al. [26], Santos et al. [28], Giles et al. [30], Doğan et al. [31], Tournadre et al. [32], and Lin et al. [33] performed cross-sectional studies, and revealed that the overall prevalence of sarcopenia was significantly higher in RA patients as compared with controls. In twelve RA studies, as highlighted in Table 1, the prevalence of sarcopenia ranged from 10.1 to 45.1% and the median value was 29.1% [26,28,30,31,32,33,34,35,36,37,38,39]. There are significant gaps among the figures. The gaps seem to result from the diversity in the definition of sarcopenia and the different features of each group such as drug use, disease activity, and ethnicity. In conclusion, RA patients are susceptible to sarcopenia, but it is difficult to determine the exact prevalence of sarcopenia in RA from the studies due to their heterogeneity.

### 3.2. Spondyloarthritis

We found three valid studies concerning the prevalence of sarcopenia in spondyloarthritis (SpA). Barone et al. studied Caucasian SpA patients aged between 40 and 75 years, excluding those with obesity; 22 patients with ankylosing spondylitis (AS) and 70 patients with PsA [39]. The prevalence of sarcopenia diagnosed using the SMI and handgrip strength was 22.7% in AS, and 20.0% in PsA [39]. The difference in the prevalence of sarcopenia between RA, PsA, and AS was not significant, whereas the prevalence of pre-sarcopenia (decreased muscle mass without reduced strength) was significantly different (As > PsA > RA) in the study [39]. In male Moroccan AS patients, the prevalence of sarcopenia was 34.3% according to the definition of the EWGSOP [41]. In another study of female patients with PsA from Poland with an age range of 50 to 75 years, the prevalence of sarcopenia was 13.7, 49.0, and 43.1%, each for different definitions [24].

### 3.3. Systemic Lupus Erythematosus

In the study by Santos et al., 16 out of 92 participants (17.4%) with a diagnosis of systemic lupus erythematosus (SLE) were sarcopenic [28]. Among them, 10.9% of patients were sarcopenic but not obese, and 6.5% patients were both sarcopenic and obese. Both numbers were significantly higher than the controls (purely sarcopenic *p* = 0.01 and sarcopenic obesity *p* = 0.009).

### 3.4. Systemic Sclerosis

Three studies calculated the prevalence of sarcopenia in systemic sclerosis (SSc). The prevalence was 20.7% when defining sarcopenia using the SMI [42] and 22.5% in a study from Germany, which included 91.5% females and followed the definition of EWGSOP [4,43]. Another study reported higher prevalence rates of 41.9 and 54.8% applying the SMI and handgrip strength criteria, respectively [44].

### 3.5. Inflammatory Bowel Disease

To estimate the degree of sarcopenia in inflammatory bowel disease (IBD), a few studies used the lumbar SMI assessed by computed tomography (CT) scan, dual-energy X-ray absorptiometry (DXA), or bioelectrical impedance analysis (BIA). Zhang et al. observed that sarcopenia was more prevalent in ulcerative colitis (UC) and Crohn’s disease (CD) as compared with controls (all *p* < 0.05) [45]. Among patients with IBD, the prevalence of sarcopenia was significantly higher in CD patients (*p* < 0.05) [45]. The prevalence of sarcopenia in UC ranged from 14.8 to 69.5% [45,47,48,50,52]. The studies used the lumbar SMI to define sarcopenia with different cut-off points. The reason for this large gap seems to result from the difference of respective inclusion criteria of the subjects. The highest prevalence of 69.5% was measured in patients who were hospitalized due to acute severe UC [47], and the lowest was in newly diagnosed patients with an age under 13 years [48]. In CD, the prevalence of sarcopenia was higher than in other autoimmune diseases. It ranged between 31.0 and 61.4% and the median was 40.2% [45,48,50,52,54,55,57,59,61,62]. There are two reasons why the numbers could have been overestimated. First, the subject groups were also skewed as described above for UC. In fact, in general, the CT data, which was used to diagnose sarcopenia, was scarce in stable patients. Therefore, subjects undergoing surgery after the CT scan were included to propose the frequency of sarcopenia [54,55], or hospitalized due to disease exacerbation [50], or suspected complications of CD [57]. Second, there could be an overlap between the two studies showing the highest prevalence because the data were measured in an identical hospital in a similar time period [45,54].

### 3.6. Other Autoimmune Diseases

The prevalence of sarcopenia in type 1 diabetes mellitus (T1DM) and latent autoimmune diabetes in adults (LADA) was 16.6 and 35.0%, respectively [63,64]. The subjects were Japanese, and sarcopenia was diagnosed according to the AWGS. In LADA, the prevalence was significantly higher than in controls [64]. Among Canadian autoimmune liver disease patients who were evaluated for liver transplantation, 41.8% of the patients were sarcopenic as diagnosed using the SMI [65].

## 4. Rheumatoid Arthritis and Sarcopenia

RA is a chronic inflammatory autoimmune disease that affects multiple synovial joints. Sarcopenia is a frequent comorbidity of RA that occurs in 10.1–45.1% of patients (Table 1). Occasionally, loss of muscle is accompanied by increased fat mass which is called sarcopenic obesity. Rheumatoid cachexia is a more serious condition and refers to the state of exhaustion and loss of overall body composition, including muscle and fat [66]. It is also a common condition in RA with a prevalence of 15–32% according to a meta-analysis [67]. Many studies have supported the idea that RA patients have lower skeletal muscle mass resulting in a higher prevalence of sarcopenia as compared with those without RA [26,31,68,69]. As shown in Table 2, sarcopenia in RA is clinically meaningful, since it is associated with the incidence of low bone mineral density, falls, and fractures [36,37]. In addition, sarcopenic RA patients have endothelial dysfunction and a higher cardiometabolic risk [34,70]. The Health Assessment Questionnaire Disability Index (HAQ-DI) is a measure to assess the functional ability of chronically ill patients, especially RA [71]. Several studies have reported that high HAQ-DI scores are associated with sarcopenia in RA [30,33,72,73]. Study findings regarding sarcopenia and RA are available in Table S1.

### 4.1. Associated Factors

The factors associated with sarcopenia in RA have been demonstrated in many studies (Table 2). Old age [36,37], BMI [34,35,36], high body fat mass [35,38], longer disease duration [37,74], bone erosion [34], low hip bone mineral density [36], malnutrition [37], low protein intake [72], and joint damage [30,33,37] were all associated with sarcopenia. Acute phase reactants such as C-reactive protein (CRP) and erythrocyte sedimentation rate (ESR) [30,36,68,72], rheumatoid factor (RF) [26,30], and matrix metalloprotease 3 (MMP3) [35] were also associated. However, conflicting results have been found for other factors. Disease activity, which was measured by the disease activity score in 28 joints (DAS28), was associated with abnormal body composition in one study [26], while others did not find a significant association [30,34,35,38]. Tada et al. stated that no significant correlation between sarcopenia and RA activity in their study could be due to the relatively mild disease activity of the subjects [35].

### 4.2. Pathogenesis

**Figure 1:**
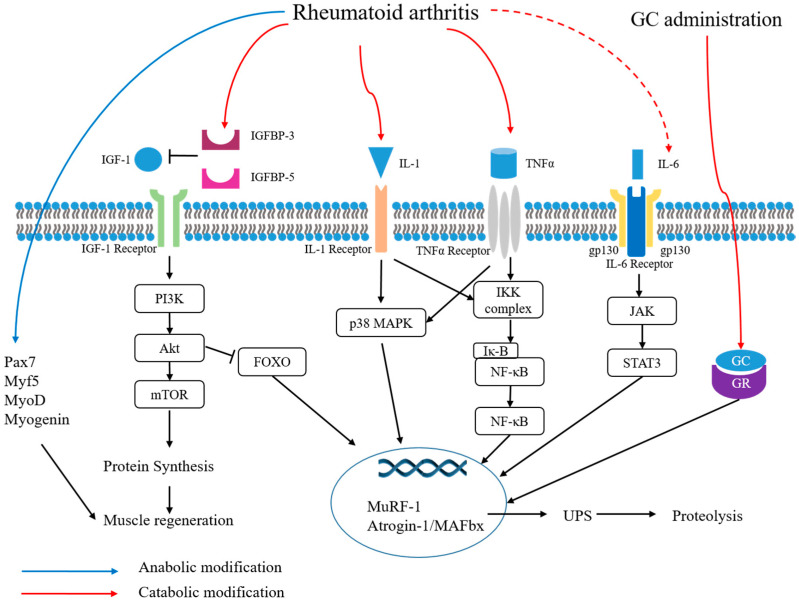
Mechanisms of sarcopenia and metabolic modifications in rheumatoid arthritis. IGF-1, insulin-like growth factor-1; IGFBP, insulin-like growth factor binding protein; IL-1, interleukin-1; TNF-α, tumor necrosis factor-α; GC, glucocorticoid; GR, glucocorticoid receptor; gp 130, glycoprotein 130; Pax7, paired box 7; PI3K, phosphoinositide 3-kinase; mTOR, mammalian target of rapamycin; MAPK, mitogen-activated protein kinase; IKK, IκB kinase; IκB, inhibitor of nuclear factor kappa B; NF-κB, nuclear factor kappa-light-chain-enhancer of activated B cells; JAK, Janus kinase; STAT3, signal transducer and activator of transcription 3; MuRF-1, muscle RING-finger 1; UPS, ubiquitin proteasome system.

Interleukin-1β (IL-1β), interleukin-6 (IL-6), and tumor necrosis factor-α (TNF-α) are proinflammatory cytokines which are thought to be pathogenic in RA. These cytokines are also associated with sarcopenia and resting energy expenditure in RA patients, as shown in Figure 1 [66,80,81]. These relationships suggest that the inflammatory response of RA promotes sarcopenia. It has been demonstrated from an animal study that muscle wasting in RA was due to the disease itself and not associated with decreased mobility [83]. The exact mechanism of muscle wasting in RA has not yet been elucidated in detail, but muscle wasting can be due to proteolysis by activated catabolic responses and not due to decreased myogenic responses [82]. In adjuvant-induced arthritis (AIA) rats, which is a model of arthritis-induced muscle wasting, increased gene expression of IL-1β accompanied with upregulation of E3 ubiquitin ligases (atrogin-1 and muscle RING-finger 1 (MuRF-1)), phosphorylated p38 mitogen-activated protein kinase (MAPK)/p38 MAPK, and active nuclear factor kappa-light-chain-enhancer of activated B cells (NF-κB) have been reported [80]. It is known that NF-κB and p38 MAPK activate the ubiquitin proteasome system [84]. These signaling pathways are related to muscle wasting in RA and they may be activated by IL-1β [80]. In contrast, myogenic regulatory factors such as MyoD, paired box 7 (Pax7), and myogenin are also increased in animals with muscle wasting [80]. These results suggest that muscle repair or anabolic compensation occur simultaneously with muscle wasting.

### 4.3. Treatments

Although available drugs for sarcopenia do not exist, it seems that treatment for RA is also helpful for RA associated sarcopenia (Table 2). Although there have been conflicting results that have indicated the use of disease-modifying antirheumatic drugs (DMARDs) was not related to changes in body composition [30], a recent study has revealed that the use of biologic DMARD was negatively associated with sarcopenia in RA [37]. A therapeutic possibility of a biologic DMARD, tocilizumab (anti-IL6 receptor antibody), has also been proposed in other studies. From a prospective study in RA patients, a year of treatment with tocilizumab increased lean mass and the SMI [32]. In addition, AIA rat studies have suggested the possibility that β2-adrenoceptor agonist (formoterol) [77], antioxidants [78], and neuromuscular electrical stimulation [79] could prevent skeletal muscle dysfunction or muscle loss in RA. In contrast, treatment of RA using glucocorticoids (GCs) seemed to exacerbate sarcopenia. It has been reported that GCs use was positively associated with low lean mass or sarcopenia in RA patients [38,72]. In a chronic polyarthritis mouse model, GCs treatment prevented inflammatory bone loss but significantly increased muscle wasting [75]. A recent study by Yamada et al. revealed that after administration of GCs for a year, 13.4% of the patients developed sarcopenia [76] and also, an average GCs use of ≥3.25 mg/day over a year was significantly associated with sarcopenia with a OR of 8.81 (95% CI 1.146–7.9, *p* = 0.037) [76]. The results imply that GC treatment in RA patients should be used cautiously and that reduction or stopping of GCs administration could alleviate treatment-related sarcopenia. However, the duration of steroid use was not associated with sarcopenia [34].

## 5. Other Rheumatic Diseases and Sarcopenia

### 5.1. Spondyloarthritis

SpA is a group of rheumatic diseases characterized by inflammation in the axial skeleton and peripheral joints, and by specific clinical symptoms such as uveitis and psoriasis [85]. SpA includes AS, PsA, and other diseases, but previous studies only investigated sarcopenia in AS and PsA. As in other rheumatic diseases, patients with SpA have been shown to be susceptible to sarcopenia [86], and it has been associated with two major factors (Table 3, Table S2). First, sarcopenia was associated with disease activity which was assessed using the Bath Ankylosing Spondylitis Disease Activity Index (BASDAI). Aguiar et al. highlighted that the SMI and the BASDAI have a significant negative correlation in male AS and PsA patients [86]. In addition, the Bath Ankylosing Spondylitis Functional Index (BASFI) was also correlated with sarcopenia in males [86]. Another study confirmed that in AS patients, sarcopenia was associated with BASDAI [41]. Second, sarcopenia was associated with bone mineral abnormality. Sarcopenic PsA patients had a significantly higher prevalence of osteoporosis than non-sarcopenic PsA patients [24]. Another study showed that sarcopenia was associated with lower bone mineral density in AS patients which supported this assumption [41]. However, other factors such as disease duration were not associated with sarcopenia.

### 5.2. Systemic Sclerosis

SSc is an autoimmune rheumatic disease characterized by vasculopathy, tissue fibrosis, and internal organ involvement [87]. SSc patients tend to have decreased muscle strength and endurance related to physical functional disability [88] and 20.7–54.8% of patients exhibit sarcopenia [42,43,44]. Sarcopenia in SSc has been associated with multiple organ involvements of the disease including lung, skin, esophagus, microvasculature, and urinary tracts (Table 3, Table S2) [42,44,87]. Among the specific characteristics of SSc, a longer duration of disease was also associated with sarcopenia [42,44,89]. In addition, low physical function [43], malnutrition [44], and high ESR [44] were also associated with sarcopenia similar to the findings in RA (Table 3). In particular, elevated ESR in SSc reflected disease severity well [90]. Considering all these results, sarcopenia seems to be related to the progression and severity of SSc and muscle weakness and atrophy could result directly from muscle involvement of SSc [91]. Thus, there could be a considerable overlap in domains of sarcopenia and muscle involvement in SSc. Interestingly, sarcopenic patients receive more immunosuppressive drugs than non-sarcopenic patients [43]. It is counterintuitive that alleviating disease activity with immunosuppressive drugs is more related to sarcopenia. Siegert et al. interpreted that receiving more drugs indicated a more severe state and a longer duration of disease [43]. Another study indicated that polypharmacy itself could directly contribute to sarcopenia [92]. However, the association between the use of multiple immunosuppressive drugs and sarcopenia needs further study. Interventional studies are still scarce but there is a single study highlighting that medical nutrition therapy reversed sarcopenia in patients with GI tract involvement of SSc [93].

**Table 3:** Associated Factors Related to Sarcopenia in Patients with Rheumatic Diseases Other Than Rheumatoid Arthritis.

**Spondyloarthritis**
BASDAI (in AS and male SpA) [41,86] BASFI (in male SpA) [86] Bone mineral density (in AS) [41] Osteoporosis (in PsA) [24]
**Systemic Sclerosis**
Lung involvement (Medsger severity score) [42] Skin involvement (mRSS, Medsger severity score) [42,44] Microvascular involvement (capillaroscopy score) [44] Esophageal involvement [44] Overactive bladder [87] Disease duration [42,44,89] DLCO [42,44] Malnutrition [44] ESR [44]

BASDAI, Bath Ankylosing Spondylitis Disease Activity Index; BASFI, Bath Ankylosing Spondylitis Function Index; mRSS, modified Rodnan skin score; DLCO, diffusing capacity for carbon monoxide; ESR, erythrocyte sedimentation rate

## 6. Inflammatory Bowel Disease and Sarcopenia

IBD includes CD and UC which are characterized by chronic relapsing bowel inflammation. The etiology of IBD remains unclear but environmental and genetic factors seem to be involved in autoimmune pathogenicity [94,95]. In IBD patients, sarcopenia is frequent and the muscle mass reduces over time accompanied by an increased BMI [96,97]. According to a follow-up study, the prevalence of sarcopenia increased from 9.3 to 16.3% until a year after the diagnosis, although sarcopenia did not increase after that time [97]. Sarcopenia in IBD has been studied for its prognostic implication and associated factors (Table S3). It has been considered to be a predictive factor for medical rescue therapy and bowel resection [47,50,52], and postoperative complications [54,62,98,99] in both CD and UC. Additionally, in CD, sarcopenia is associated with primary non-response to anti-TNF treatment, and therefore sarcopenic IBD patients need adjusted dosing [100]. The mechanism of sarcopenia in IBD patients is believed to be associated with disease-related inflammation and nutritional problems. Muscle radiation attenuation, which is an inverse parameter of muscle fat content [101], has been associated with severe phenotypes of disease such as a history of a stricturing, penetrating complication, or previous resection surgery for CD [61]. In addition, sarcopenia has been associated with high disease activity assessed by the Mayo score in UC [45]. Inflammatory markers such as CRP and ESR, have been associated with sarcopenia in IBD [59,99]. In addition, vitamin D in pediatric patients, as well as hemoglobin and albumin in adult patients have been associated with sarcopenia [48,59]. Decreased motility also seems to contribute to sarcopenia in pediatric patients [102]. Additionally, we suggest a possibility that the gut microbiome could be related to sarcopenia in IBD. In IBD, the composition and function of microbiome are altered. It has been reported that IBD patients have increased proinflammatory bacterial species (Escherichia, Fusobacterium) and decreased anti-inflammatory bacterial species (Faecalibacterium) with decreased amino acid biosynthesis of the microbiome [103]. In addition, it has been suggested that the gut microbiome could directly affect the muscle by modulating amino acid bioavailability and the production of proinflammatory cytokines [104]. In an acute leukemia mouse model, oral supplementation of lactobacillus species decreased atrogin-1, MuRF1, and inflammatory cytokines [105]. A direct association of muscle and gut microbiome in sarcopenic IBD should be investigated by animal and clinical studies. To alleviate sarcopenia in IBD, treatment of the disease through reduction in inflammation would be effective. Infliximab, a TNF-α antibody, increased both muscle volume and strength in CD patients [106], and moreover, colectomy increased SMI and serum albumin with a decrease in the prevalence of sarcopenia in UC patients [45]. Nutritional management could also be needed for better postoperative prognosis in sarcopenic IBD patients, although it is not effective directly in the management of sarcopenia [54].

## 7. Autoimmune Diabetes and Sarcopenia

T1DM is a chronic autoimmune disease characterized by hyperglycemia due to pancreatic islet β-cell destruction [107]. T1DM patients have a high prevalence of sarcopenia and hyperglycemia is linked with low muscle function [63,108]. There are many factors that contribute to muscle dysfunction in diabetes. Excessive intramyocellular lipid (IMCL) lowers muscle quality and could impair muscle function [109]. Increased IMCL is frequently observed in T1DM patients [110]. Especially, increased IMCL is associated with poor glycemic control evaluated by hemoglobin A1c (HbA1c) [111]. Accumulation of advanced glycation end-products, which are associated with persistent hyperglycemia [112], is also thought to contribute to low muscle function in T1DM patients [63]. In addition, it has been reported that hyperglycemia is linked with muscle atrophy via a WW domain containing E3 ubiquitin protein ligase 1 (WWP1)/Krüppel-like factor 15 (KLF15) pathway [113]. Hyperglycemia inhibits degradation of KLF15 via downregulation of WWP1 and increased KLF15 promotes proteolysis via upregulation of atrogin-1 and MuRF1 [113,114]. Moreover, hormones or cytokines that are related to skeletal muscle are altered in T1DM. Diabetic patients appear to have higher GC and IL-6 levels and both have catabolic effects [115,116,117]. Moreover, insulin-like growth factor-1 (IGF-1) which is well known for its contribution to skeletal muscle regeneration and development is decreased with an alteration of the IGFBP [118,119,120]. Recently, mitochondrial dysfunction in T1DM has been suggested as a primary contributor to muscle dysfunction. Mitochondrial changes in T1DM-related sarcopenia are similar to that in age-related ones, and both include elevated oxidative stress and mitochondrial-induced cell death [121]. LADA is a subtype of T1DM but has insulin resistance similar to type 2 diabetes mellitus (T2DM) [122]. LADA had a higher risk of sarcopenia as compared with controls and even T2DM groups in a cross-sectional study, but, so far, the association between LADA and sarcopenia has not been elucidated in great detail [64].

## 8. Conclusions

In this in-depth review, we provide evidence that sarcopenia is common in different autoimmune and rheumatic diseases. The exact prevalence differs among different studies, in part, due to the different definitions of sarcopenia that are used. We propose that reporting sarcopenia in autoimmune and rheumatic disorders is essential, since it contributes to morbidity and mortality among these patients. Specific risk factors need to be confirmed in larger studies with a particular focus on treatment strategies, i.e., cumulative dose of GC or other immunosuppressive measures. More detailed analyses highlighting the role of chronic inflammation in the propagation of sarcopenia are needed.

## Figures and Tables

**Table 1:** Prevalence of Sarcopenia in Patients with Autoimmune and Rheumatic Diseases.

Author	Prevalence (%)		Patients (N)	Group Feature	*p*-Value	Definition of Sarcopenia (Cut-off)
**Rheumatoid Arthritis**						
Dao et al. [26] ^1^	Purely sarcopenic	18.1	105	Vietnamese, female	0.007	FFMI (Hull et al. [27])
	Sarcopenic obesity ^2^	12.4			0.002	
	Total	30.5			-	
Santos et al. [28] ^1^	Purely sarcopenic	4.5	89	Caucasian, Portuguese, female	>0.05 ^3^	FFMI z score ≤ −2 (Schutz et al. [29])
	Sarcopenic obesity ^2^	5.6			0.01	
	Total	10.1			-	
Giles et al. [30]	Male	33.3	72	American	0.157 ^3^	SMI (Janssen et al. [14])
	Female	21.4	117		0.004	
	Total	25.9	189		-	
Doğan et al. [31]	43.3		30	Female, Age 35–50	0.004	SMI (Janssen et al. [14])
Tournadre et al. [32]	28.6		21	Active RA (DAS28 > 3.2)	<0.05	SMI (Baumgartner et al. [13])
Lin et al. [33]	45.1		457	Chinese	<0.05 ^4^	SMI (AWGS [18])
Ngeuleu et al. [34]	39.8		123	Moroccan	-	SMI (Baumgartner et al. [13])
Tada et al. [35]	28.0		100	Japanese	-	AWGS [18]
Mochizuki et al. [36]	29.6		240	Japanese, age ≥ 65	-	AWGS [18]
Torii et al. [37]	37.1		388	Japanese, female	-	EWGSOP [4], AWGS [18]
Vlietstra et al. [38]	17.1		82	New Zealander	-	SMI (FNIH [15])
Barone et al. [39]	21.0		76	Caucasian, Italian, age 40–75	-	SMI (Janssen et al. [14]), HS (Lauretani et al. [40])
**Spondyloarthritis** **Ankylosing Spondylitis**						
Barone et al. [39]	22.7		22	Caucasian, Italian, age 40–75	-	SMI (Janssen et al. [14]), HS (Lauretani et al. [40])
El Maghraoui et al. [41]	34.3		67	Moroccan, male	-	EWGSOP [4]
**Psoriatic Arthritis**						
Barone et al. [39]	20.0		70	Caucasian, Italian, age 40–75	-	SMI (Janssen et al. [14]), HS (Lauretani et al. [40])
Krajewska-Włodarczyk et al. [24]	13.7		51	Polish, age 50–75, female	-	SMI (Baumgartner et al. [13])
	49.0					SMI (Janssen et al. [25])
	43.1					SMI(Janssen et al. [25]), TUG > 14s
**Systemic Lupus Erythematosus**						
Santos et al. [28] ^1^	Purely sarcopenic	10.9	92	Caucasian, Portuguese, female	0.01	FFMI (Schutz et al. [29])
	Sarcopenic obesity ^2^	6.5			0.009	
	Total	17.4			-	
**Systemic Sclerosis**						
Caimmi et al. [42]	20.7		140	Italian	-	SMI (Baumgartner et al. [13])
Siegert et al. [43]	22.5		129	German, 91.5% female	-	EWGSOP [4]
Corallo et al. [44]	41.9		62	Caucasian, Italian	-	SMI (Baumgartner et al. [13])
	54.8					HS (Male < 30, Female < 20)
**Inflammatory Bowel Disease** **Ulcerative colitis**						
Zhang et al. [45]	27.3		99	Chinese.	<0.05	SMI (Fearon et al. [46])
Cushing et al. [47]	69.5		82	Admitted for ASUC	-	SMI (Fearon et al. [46])
Mager et al. [48]	14.8		27	Age 5–18	-	SMM z score < −2 [49]
Bamba et al. [50]	48.3		29	Japanese	-	SMI (Nishikawa et al. [51])
Adams et al. [52]	50.0		14	American	-	SMI (Prado et al. [53])
**Crohn’s Disease**						
Zhang et al. [45]	59.0		105	Chinese	<0.05	SMI (Fearon et al. [46])
Mager et al. [48]	31.0		58	Age 5–18	-	SMM z score < −2 [49]
Zhang et al. [54]	61.4		114	Chinese, required BR	-	SMI (Fearon et al. [46])
O’Brien et al. [55]	39.0		77	Retrospectively selected (BR)	-	SMI (Martin et al. [56])
Bamba et al. [50]	37.2		43	Japanese	-	SMI (Nishikawa et al. [51])
Thiberge et al. [57]	33.6		149	French	-	SMI (Mourtzakis et al. [58])
Adams et al. [52]	44.7		76	American	-	SMI (Prado et al. [53])
Lee et al. [59]	50.6		79	Korean	-	SMI (Kim et al. [60])
Cravo et al. [61]	31.0		71	Portuguese	-	SMI (Martin et al. [56])
Carvalho et al. [62]	41.4		58	Portuguese	-	SMI (Prado et al. [53])
**Diabetes** **Type 1 Diabetes Mellitus**						
Mori et al. [63]	16.6		36	Japanese	-	AWGS [18]
**Latent Autoimmune Diabetes in Adults**						
Bouchi et al. [64]	35.0		20	Japanese	0.022	AWGS [18]
**Autoimmune Liver Disease** **(Autoimmune Hepatitis, Primary Biliary Cirrhosis, Primary Sclerosing Cholangitis)**						
Montano-Loza et al. [65]	41.8		55	Canadian, evaluated for LT	-	SMI (Martin et al. [56])

FFMI, free fat mass index; SMI, skeletal muscle mass index; RA, rheumatoid arthritis; DAS28, disease activity score in 28 joints; HS, handgrip strength; TUG, timed up and go; ASUC, acute severe ulcerative colitis; SMM, skeletal muscle mass; BR, bowel resection; LT, liver transplantation. ^1^ Sarcopenia was divided into two groups, purely sarcopenic and sarcopenic obesity; ^2^ Sarcopenic obesity refers to a medical condition in which the loss of muscle is accompanied by increased fat mass; ^3^ Not statistically significant; ^4^ *p*-value was measured respectively according to sex and age. Each *p*-value was <0.05.

**Table 2:** Study Findings Related to Sarcopenia in Patients with Rheumatoid Arthritis.

Associated Factors
Age [36,37] BMI [34,35,36] Body fat mass [35,38] Disease duration [37,74] Bone erosion and mineral density [34,36] Malnutrition and protein intake [37,72] Joint damage [30,33,37] Functional status (HAQ score) [26,30,33,72,73] CRP level [30,36,68,72] ESR [68,72] RF [26,30] MMP3 [35] Use of GC [38,72,75,76]
Treatment
IL-6 inhibitor (TCZ) [32] DMARDs [30,37] β_2_-adrenoceptor agonist (formoterol) [77] Antioxidant [78] Neuromuscular electrical stimulation [79]
Risk
Falls [37] Fractures [37] Low bone mineral density [37] Cardiometabolic risk [34] Endothelial dysfunction [70]
Cytokines/Pathways
IL-1β [66,80] IL-6 [81] TNF- α [66,81] NF-Κb [80] p38 MAPK [80] pSTAT3 [80] Pax7 [80] Myostatin [80] MyoD [80,82] Myogenin [80,82] IGFBP-5 [82] IGFBP-3 [82] atrogin-1 [80,82] MuRF-1 [80,82]

BMI, body mass index; HAQ, health assessment questionnaire; CRP, C-reactive protein; ESR, erythrocyte sedimentation rate; RF, rheumatoid factor; MMP3, matrix metallopeptidase 3; GC, glucocorticoid; IL-6, interleukin-6; TCZ, tocilizumab; DMARDs, disease-modifying antirheumatic drugs; IL-1β, interleukin-1β; TNF-α, tumor necrosis factor-α; NF-κB, nuclear factor kappa-light-chain-enhancer of activated B cells; MAPK, mitogen-activated protein kinase; pSTAT3, phospho-signal transducer and activator of transcription 3; Pax7, paired box 7; IGFBP, insulin-like growth factor binding protein; MuRF-1, muscle RING-finger 1.

## Supplementary Materials

Supplementary materials can be found at https://www.mdpi.com/1422-0067/21/16/5678/s1, Table S1: Study findings of rheumatoid arthritis and sarcopenia, Table S2: Study findings of rheumatic diseases other than rheumatoid arthritis and sarcopenia, Table S3: Study findings of inflammatory bowel disease and sarcopenia.

## Abbreviations

ICD	International Classification of Diseases
OR	Odds ratio
RA	Rheumatoid arthritis
SMI	Skeletal muscle mass index
ASM	Appendicular skeletal muscle mass
EWGSOP	The European Working Group on Sarcopenia in Older People
FNIH	The Foundation of the National Institute of Health
IWGS	The International Working Group on Sarcopenia
ESPEN SIG	The European Society on Clinician Nutrition and Metabolism special interest groups
AWGS	The Asian Working Group for Sarcopenia
EWGSOP2	The European Working Group on Sarcopenia in Older People 2
FFMI	Free fat mass index
PsA	Psoriatic arthritis
TUG	Timed up and go
SpA	Spondyloarthritis
AS	Ankylosing spondylitis
SLE	Systemic lupus erythematosus
SSc	Systemic sclerosis
IBD	Inflammatory bowel disease
CT	Computed tomography
UC	Ulcerative colitis
CD	Crohn’s disease
T1DM	Type 1 diabetes mellitus
LADA	Latent autoimmune diabetes in adults
HAQ-DI	Health Assessment Questionnaire Disability Index
CRP	C-reactive protein
ESR	Erythrocyte sedimentation rate
IL-1β	Interleukin-1β
IL-6	Interleukin-6
TNF-α	Tumor necrosis factor-α
AIA	Adjuvant-induced arthritis
MuRF-1	Muscle RING-finger 1
MAPK	Mitogen-activated protein kinase
NF-κB	Nuclear factor kappa-light-chain-enhancer of activated B cells
Pax-7	Paired box 7
DMARD	Disease-modifying antirheumatic drug
GC	Glucocorticoid
BASDAI	Bath Ankylosing Spondylitis Disease Activity Index
BASFI	Bath Ankylosing Spondylitis Functional Index
IMCL	Intramyocellular lipid
WWP1	WW domain containing E3 ubiquitin protein ligase 1
KLF15	Krüppel-like factor 15
IGF-1	Insulin-like growth factor-1
IGFBP	Insulin-like growth factor binding protein
T2DM	Type 2 diabetes mellitus
