# Supplementary material for: Sarcopenia in Autoimmune and Rheumatic Diseases: A Comprehensive Review
Source: Int J Mol Sci. 2020 Aug 7;21(16):5678. doi: 10.3390/ijms21165678 (PMC7461030; doi:10.3390/ijms21165678)
Supplement: Supplementary file 1 [file ijms-21-05678-s001.pdf]

International Journal of
Molecular Sciences

MDPI

Supplemental tables

Sarcopenia in Autoimmune and Rheumatic Diseases: A Comprehensive Review

Table S1. Study findings of rheumatoid arthritis and sarcopenia
Table S2. Study findings of rheumatic diseases other than rheumatoid arthritis and sarcopenia
Table S3. Study findings of inflammatory bowel disease and sarcopenia

Table 1. Study Findings of Rheumatoid Arthritis and Sarcopenia.

Author	Study Findings	Definition of Sarcopenia
	Vulnerability	
Dao et al. [1]	Female early RA patients in Vietnam had lower appendicular LM.	FFMI (Hull et al. [2])
Doğan et al. [3]	Female RA patients had lower SMI and higher sarcopenia prevalence.	SMI (Janssen et al. [4])
Santo et al. [5]	According to meta-analysis, rheumatoid cachexia is a common comorbidity in RA whose prevalence is 15-32%.	-
Munro et al. [6]	RA patients had lower upper arm muscle mass.	-
Kasher et al. [7]	Female RA patients in Kazakhstan had lower MMI.	-
	Associated factors	
Dao et al. [1]	Disease activity, functional status, RF seropositivity was associated with abnormal body composition in female RA patients.	FFMI (Hull et al. [2])
Giles et al. [8]	CRP levels, RF seropositivity, joint deformity, functional limitation was associated with abnormal body composition in RA patients.	SMI (Janssen et al. [4])
Ngeuleu et al. [9]	Bone erosion, normal/over fat BMI were associated to sarcopenia but disease activity and functional status were not associated in RA patients.	SMI (Baumgartner et al. [10])
Lin et al. [11]	Functional limitation (HAQ-DI > 1) and joint damage were positively associated with sarcopenia in RA patients.	SMI (AWGS [12])
Tada et al. [13]	BMI, body fat mass, and MMP3 were associated with sarcopenia in RA patients.	AWGS [12]
Mochizuki et al. [14]	Age, BMI, CRP, hip bone mineral density were significantly associated with sarcopenia in RA patients.	AWGS [12]
Torii et al. [15]	Age, longer disease duration, joint destruction and malnutrition were associated with sarcopenia in RA patients.	EWGSOP [16], AWGS [12]
Vlietstra et al. [17]	Higher body fat was associated with sarcopenia but self-reported fatigue and physical function were not associated in RA patients.	SMI (FNIH [18])
Munro et al. [6]	The acute phase response (ESR, CRP) had a significant correlation with reduced fat free mass in female RA patients.	-
Müller et al. [19]	Higher ESR, CRP, lower protein intake, worse functional status were associated with having low lean mass in early RA patients.	ALM/height^2 (male< 8.0586 kg/h^2 female< 6.0359 kg/h^2)

Alkan Melikoğlu [20]	SMI of RA patients had a negative correlation with functional status.	-
Beenakker et al. [21]	The low handgrip strength was negatively associated with disease duration but not associated with age between 35 and 65 years in RA patients.	-
	Drugs/Treatment	
Giles et al. [8]	Not using DMARDs was associated with abnormal body composition in RA patients.	SMI (Janssen et al. [4])
Ngeuleu et al. [9]	There was no significant difference according to duration of steroid use between sarcopenic and non-sarcopenic RA group.	SMI (Baumgartner et al. [10])
Tournadre et al. [22]	TCZ was effective in gain of weight, lean mass, appendicular lean mass and SMI without fat mass gain in RA patients.	SMI (Baumgartner et al. [10])
Torii et al. [15]	Use of bDMARDs was negatively associated with sarcopenia in RA patients.	EWGSOP [16], AWGS [12]
Vlietstra et al. [17]	GC use was associated with sarcopenia in RA patients.	SMI (FNIH [18])
Müller et al. [19]	Current GC use was associated with having low lean mass in the early RA group.	ALM/height^2 (male< 8.0586 kg/h^2 female< 6.0359 kg/h^2)
Gómez-SanMiguel et al. [23]	In AiA rats, formoterol administration decreased severity of disease and skeletal muscle loss. It was associated with decreased inflammation, myostatin, the p-NF-κB(p65)/TNF pathway, IGFBP-3 and increased Akt and myogenin.	-
Yamada et al. [24]	According to an animal study of AiA rats, antioxidant treatment could prevent skeletal muscle dysfunction in RA patients.	-
Himori et al. [25]	According to an animal study of AiA rats, neuromuscular electrical stimulation could prevent skeletal muscle dysfunction in RA patients.	-
Fenton et al. [26]	In mice models of chronic polyarthritis, GC increased muscle wasting but reduced bone loss.	-
Yamada et al. [27]	GC use could promote sarcopenia in RA patients.	AWGS [12]
	Risk	
Ngeuleu et al. [9]	Sarcopenia was associated with cardiometabolic risk in RA patients	SMI (Baumgartner et al. [10])
Torii et al. [15]	The incidence of falls, fractures, and lower bone mineral density were higher in patients with sarcopenia.	EWGSOP [16], AWGS [12]

Delgado-Frías et al. [28]	Sarcopenia in RA patients was associated with lower endothelial function.	SMI (Janssen et al. [4])
	Mechanism	
Roubenoff et al. [29]	Loss of body cell mass, high TNF-α and IL-1 were observed and cytokine production was associated with resting energy expenditure in RA patients.	-
Little et al. [30]	In AiA rabbits, reduction of muscle mass and diameter seem to be related with increased IL-1β, NF-κB, p38 MAPK signaling and seem to trigger anabolic compensation of increased myonuclei, Pax7, MyoD, myogenin and reduced pSTAT3, myostatin.	-
Visser et al. [31]	Higher plasma concentrations of IL-6 and TNF-α were associated with lower muscle mass and muscle strength.	-
de Oliveira Nunes Teixeira et al. [32]	In a CIA rat study, muscle atrophy was not associated with decreased mobility.	-
Castillero et al. [33]	In AiA rats, arthritis-induced skeletal muscle atrophy may be due to proteolysis resulting from increased IGFBP-5, IGFBP-3, atrogin-1 and MuRF-1 but not from a decrease in the myogenic regulatory factors.	-

LM, lean mass; FFMI, free fat mass index; SMI, skeletal muscle mass index; MMI, muscle mass index; RF, rheuatoid factor; CRP, C-reactive protein; BMI, body mass index; HAQ-DI, health assessment questionnaire disability index; MMP3, matrix metallopeptidase 3; ESR, erythrocyte sedimentation rate; ALM, appendicular lean mass; DMARD, disease-modifying antirheumatic drug; TCZ, tocilizumab; bDMARD, biologic disease-modifying antirheumatic drug; GC, glucocorticoid; AiA, adjuvant-induced arthritis; NF-κB, nuclear factor kappa-light-chain-enhancer of activated B cells; TNF, tumor necrosis factor; IGFBP, insulin-like growth factor-binding protein; IL-1, interleukin-1; MAPK, mitogen-activated protein kinase; Pax7, paired box 7; pSTAT3, phospho-signal transducer and activator of transcription 3; IL-6, interleukin-6; CIA, collagen-induced arthritis; MuRF-1, muscle RING finger 1.

Table 2. Study Findings of Rheumatic Diseases Other Than Rheumatoid Arthritis and Sarcopenia.

Author	Findings	Definition of Sarcopenia
	Spondyloarthritis	
El Maghraoui et al. [34]	Sarcopenia and pre-sarcopenia associated with high BASDAI and low BMD in AS patients.	EWGSOP [16]
Krajewska-Włodarczyk et al. [35]	Sarcopenia was associated with occurrence of osteoporosis in PsA patients.	SMI (Janssen et al. [36]), TUG>14s
Aguiar et al. [37]	SMI was correlated with BASDAI and BASFI in male patients.	SMI (Male: <10.75, Female: 6.75)
	Systemic sclerosis	
Caimmi et al. [38]	Sarcopenia was associated with longer disease duration, worse DLCO/VA, lung and skin involvement (Medsger severity score).	SMI (Baumgartner et al. [10])
Siegert et al. [39]	Sarcopenic patients had lower physical function and more immunosuppressive drugs than non-sarcopenic patients.	EWGSOP [16]
Corallo et al. [40]	When defining sarcopenia according to SMI and HS separately, both were associated with malnutrition, disease duration, mRSS, capillaroscopy score, esophageal involvement, ESR and DLCO.	SMI (Baumgartner et al. [10]) or HS (Male: <30, Female:<20)
Pacini et al. [41]	Sarcopenia is positively correlated with overactive bladder.	SMI (Baumgartner et al. [10])
Justo et al. [42]	Female SSc patients had reduced muscle strength, endurance and it was correlated with physical disability.	-
Marighela et al. [43]	Longer disease duration was correlated with SMI.	SMI (Baumgartner et al. [10])
Doerfler et al. [44]	Medical nutrition therapy intervention reversed sarcopenia in GI involvement patients.	SMI (Baumgartner et al. [10])

BASDAI, Bath Ankylosing Spondylitis Disease Activity; BMD, bone mineral density; BASDFI, Bath Ankylosing Spondylitis Function Index; DLCO, diffusing capacity for carbon monoxide; VA, alveolar volume; mRSS, modified Rodnan Skin Score; GI, gastrointestinal.

Table 3. Study Findings of Inflammatory Bowel Disease and Sarcopenia.

Author	Findings	Definition of Sarcopenia
	Inflammatory Bowel Disease (no separation into CD and UC)	
Adams et al. [45]	Sarcopenia is a predictor of need for surgery.	SMI (Prado et al. [46])
Bryant et al. [47]	SMI continuously decreased over time in newly diagnosed IBD patients, while sarcopenia did not.	SMI, HS (EWGSOP [16])
Pedersen et al. [48]	Sarcopenia is a predictor of postoperative complications in patients younger than 40 years.	Lowest sex quartile of TPI or HUAC.
Werkstetter et al. [49]	Pediatric IBD patients have lower lean body mass, muscle strength and reduced physical activity.	-
	Crohn's Disease	
Mager et al. [50]	In children with IBD, sarcopenia is more prevalent in CD than UC.<br>Sarcopenia is associated with suboptimal vitamin D levels (<50 nmol/l) in CD.	SMM z score<-2 [51]
Zhang et al. [52]	Sarcopenia is a predictor of major postoperative complications (grade ≥ III on the Clavien-Dindo scale[53]).	SMI (Fearon et al. [54])
Bamba et al. [55]	Sarcopenia is a predictor of need for surgery.	SMI (Nishikawa et al. [56])
Thiberge et al. [57]	SMI was non-significantly lower in the group of patients needing surgery or dying during follow-up.	SMI (Mourtzakis et al. [58])
Lee et al. [59]	CRP was associated with sarcopenia and ESR, serum albumin, hemoglobin were correlated with SMI.	SMI (Kim et al. [60])
Cravo et al. [61]	Lower muscle attenuation was associated with severe phenotypes (stricturing or penetrating)	SMI (Martin et al. [62])
Carvalho et al. [63]	Sarcopenia is a predictor of postoperative complications.	SMI (Prado et al. [46])
Ding et al. [64]	Sarcopenia was associated with primary nonresponse to anti-TNF therapy	Lowest sex quartile of SMI.
Subramaniam et al. [65]	Infliximab increased muscle volume and strength.	-

Ulcerative Colitis		
Zhang et al. [66]	Sarcopenia was associated with high disease activity (high Mayo score) and colectomy reversed sarcopenia.	SMI (Fearon et al. [54])
Cusing et al. [67]	Sarcopenia is a predictor of need for medical rescue therapy or surgery in hospitalized ASUC patients.	SMI (Fearon et al. [54])
Fujikawa et al. [68]	Sarcopenia is a predictor of surgical site infection.<br>Sarcopenia is associated with CRP.	Lowest sex quartile of TPI.

TPI, total psoas index; HUAC, Hounsfield unit average calculations; ASUC, acute severe ulcerative colitis.

References

1. Dao, H.H.; Do, Q.T.; Sakamoto, J. Abnormal body composition phenotypes in Vietnamese women with early rheumatoid arthritis. *Rheumatology (Oxford).* **2011**, *50*, 1250–1258, doi:10.1093/rheumatology/ker004.
2. Hull, H.R.; Thornton, J.; Wang, J.; Pierson, R.N.; Kaleem, Z.; Pi-Sunyer, X.; Heymsfield, S.; Albu, J.; Fernandez, J.R.; Vanitallie, T.B.; et al. Fat-free mass index: Changes and race/ethnic differences in adulthood. *Int. J. Obes.* **2011**, *35*, 121–127, doi:10.1038/ijo.2010.111.
3. Ceyhan Dogan, S.; Hizmetli, S.; Hayta, E.; Kaptanoglu, E.; Erselcan, T.; Guler, E. Sarcopenia in women with rheumatoid arthritis. *Eur. J. Rheumatol.* **2015**, *2*, 57–61, doi:10.5152/eurjrheum.2015.0038.
4. Janssen, I.; Baumgartner, R.N.; Ross, R.; Rosenberg, I.H.; Roubenoff, R. Skeletal muscle cutpoints associated with elevated physical disability risk in older men and women. *Am J Epidemiol* **2004**, *159*, 413–421, doi:10.1093/aje/kwh058.
5. Santo, R.C.E.; Fernandes, K.Z.; Lora, P.S.; Filippin, L.I.; Xavier, R.M. Prevalence of rheumatoid cachexia in rheumatoid arthritis: a systematic review and meta-analysis. *J. Cachexia. Sarcopenia Muscle* **2018**, *9*, 816–825, doi:10.1002/jcsm.12320.
6. Munro, R.; Capell, H. Prevalence of low body mass in rheumatoid arthritis: Association with the acute phase response. *Ann. Rheum. Dis.* **1997**, *56*, 326–329, doi:10.1136/ard.56.5.326.
7. Kasher, M.; Gabdulina, G.; Beissebayeva, A.; Mussabaeva, D.; Tokarev, A.; Sarssenbayeva, M.; Omarova, K.; Mominova, G.; Livshits, G. Rheumatoid arthritis is associated with exacerbated body composition deterioration in Kazakh females. *Nutrition* **2019**, *66*, 219–226, doi:10.1016/j.nut.2019.06.007.
8. Giles, J.T.; Ling, S.M.; Ferrucci, L.; Bartlett, S.J.; Andersen, R.E.; Towns, M.; Muller, D.; Fontaine, K.R.; Bathon, J.M. Abnormal body composition phenotypes in older rheumatoid arthritis patients: Association with disease characteristics and pharmacotherapies. *Arthritis Care Res.* **2008**, *59*, 807–815, doi:10.1002/art.23719.
9. Ngeuleu, A.; Allali, F.; Medrare, L.; Madhi, A.; Rkain, H.; Hajjaj-Hassouni, N. Sarcopenia in rheumatoid arthritis: prevalence, influence of disease activity and associated factors. *Rheumatol. Int.* **2017**, *37*, 1015–1020, doi:10.1007/s00296-017-3665-x.

10. Baumgartner, R.N.; Koehler, K.M.; Gallagher, D.; Romero, L.; Heymstleld, S.B.; Ross, R.R.; Garry, P.J.; Lindeman, R.D. Epidemiology of Sarcopenia among the Elderly in New Mexico. *Am J Epidemiol* **1998**, *147*, 755–763, doi:10.1093/oxfordjournals.aje.a009520.
11. Lin, J.Z.; Liang, J.J.; Ma, J. Da; Li, Q.H.; Mo, Y.Q.; Cheng, W.M.; He, X.L.; Li, N.; Cao, M.H.; Xu, D.; et al. Myopenia is associated with joint damage in rheumatoid arthritis: a cross-sectional study. *J. Cachexia. Sarcopenia Muscle* **2019**, *10*, 355–367, doi:10.1002/jcsm.12381.
12. Chen, L.K.; Liu, L.K.; Woo, J.; Assantachai, P.; Auyeung, T.W.; Bahyah, K.S.; Chou, M.Y.; Chen, L.Y.; Hsu, P.S.; Krairit, O.; et al. Sarcopenia in Asia: Consensus report of the Asian working group for sarcopenia. *J. Am. Med. Dir. Assoc.* **2014**, *15*, 95–101, doi: 10.1016/j.jamda.2013.11.025.
13. Tada, M.; Yamada, Y.; Mandai, K.; Hidaka, N. Matrix metalloprotease 3 is associated with sarcopenia in rheumatoid arthritis - results from the CHIKARA study. *Int. J. Rheum. Dis.* **2018**, *21*, 1962–1969, doi:10.1111/1756-185X.13335.
14. Mochizuki, T.; Yano, K.; Ikari, K.; Okazaki, K. Sarcopenia-associated factors in Japanese patients with rheumatoid arthritis: A cross-sectional study. *Geriatr. Gerontol. Int.* **2019**, *19*, 907–912, doi:10.1111/ggi.13747.
15. Torii, M.; Hashimoto, M.; Hanai, A.; Fujii, T.; Furu, M.; Ito, H.; Uozumi, R.; Hamaguchi, M.; Terao, C.; Yamamoto, W.; et al. Prevalence and factors associated with sarcopenia in patients with rheumatoid arthritis. *Mod. Rheumatol.* **2019**, *29*, 589–595, doi:10.1080/14397595.2018.1510565.
16. Cruz-Jentoft, A.J.; Baeyens, J.P.; Bauer, J.M.; Boirie, Y.; Cederholm, T.; Landi, F.; Martin, F.C.; Michel, J.P.; Rolland, Y.; Schneider, S.M.; et al. Sarcopenia: European consensus on definition and diagnosis. *Age Ageing* **2010**, *39*, 412–423, doi:10.1093/ageing/afq034.
17. Vlietstra, L.; Stebbings, S.; Meredith-Jones, K.; Haxby Abbott, J.; Treharne, G.J.; Waters, D.L. Sarcopenia in osteoarthritis and rheumatoid arthritis: The association with self-reported fatigue, physical function and obesity. *PLoS One* **2019**, *14*, e0217462, doi:10.1371/journal.pone.0217462.
18. Studenski, S.A.; Peters, K.W.; Alley, D.E.; Cawthon, P.M.; McLean, R.R.; Harris, T.B.; Ferrucci, L.; Guralnik, J.M.; Fragala, M.S.; Kenny, A.M.; et al. The FNIH sarcopenia project: rationale, study description, conference recommendations, and final estimates. *J. Gerontol. A. Biol. Sci. Med. Sci.* **2014**, *69*, 547–558, doi:10.1093/gerona/glu010.
19. Müller, R.; Kull, M.; Põlluste, K.; Valner, A.; Lember, M.; Kallikorm, R. Factors associated with low lean mass in early rheumatoid arthritis: A cross-sectional study. *Med.* **2019**, *55*, 730, doi:10.3390/medicina55110730.
20. Alkan Melıkoğlu, M. Presarcopenia and its impact on disability in female patients with rheumatoid arthritis. *Arch. Rheumatol.* **2017**, *32*, 53–59, doi:10.5606/ArchRheumatol.2017.6078.
21. Beenakker, K.G.M.; Ling, C.H.; Meskers, C.G.M.; de Craen, A.J.M.; Stijnen, T.; Westendorp, R.G.J.; Maier, A.B. Patterns of muscle strength loss with age in the general population and patients with a chronic inflammatory state. *Ageing Res. Rev.* **2010**, *9*, 431–436, doi: 10.1016/j.arr.2010.05.005.
22. Tournadre, A.; Pereira, B.; Dutheil, F.; Giraud, C.; Courteix, D.; Sapin, V.; Frayssac, T.; Mathieu, S.; Malochet-Guinamand, S.; Soubrier, M. Changes in body composition and metabolic profile during interleukin 6 inhibition in rheumatoid arthritis. *J. Cachexia. Sarcopenia Muscle* **2017**, *8*, 639–646, doi:10.1002/jcsm.12189.
23. Gómez-SanMiguel, A.B.; Gomez-Moreira, C.; Nieto-Bona, M.P.; Fernández-Galaz, C.; Villanúa, M.Á.; Martín, A.I.; López-Calderón, A. Formoterol decreases muscle wasting as well as inflammation in the rat model of rheumatoid arthritis. *Am. J. Physiol. - Endocrinol. Metab.* **2016**, *310*, E925–E937, doi:10.1152/ajpendo.00503.2015.
24. Yamada, T.; Abe, M.; Lee, J.; Tatebayashi, D.; Himori, K.; Kanzaki, K.; Wada, M.; Bruton, J.D.; Westerblad, H.; Lanner, J.T. Muscle dysfunction associated with adjuvant-induced arthritis is prevented by antioxidant treatment. *Skelet. Muscle* **2015**, *5*, 20, doi:10.1186/s13395-015-0045-7.
25. Himori, K.; Tatebayashi, D.; Kanzaki, K.; Wada, M.; Westerblad, H.; Lanner, J.T.; Yamada, T. Neuromuscular electrical stimulation prevents skeletal muscle dysfunction in adjuvant-induced arthritis rat. *PLoS One* **2017**, *12*, e0179925, doi:10.1371/journal.pone.0179925.
26. Fenton, C.G.; Webster, J.M.; Martin, C.S.; Fareed, S.; Wehmeyer, C.; MacKie, H.; Jones, R.; Seabright, A.P.; Lewis, J.W.; Lai, Y.C.; et al. Therapeutic glucocorticoids prevent bone loss but drive muscle wasting when administered in chronic polyarthritis. *Arthritis Res. Ther.* **2019**, *21*, 182, doi:10.1186/s13075-019-1962-3.

27. Yamada, Y.; Tada, M.; Mandai, K.; Hidaka, N.; Inui, K.; Nakamura, H. Glucocorticoid use is an independent risk factor for developing sarcopenia in patients with rheumatoid arthritis: from the CHIKARA study. *Clin. Rheumatol.* **2020**, doi:10.1007/s10067-020-04929-4.
28. Delgado-Frías, E.; González-Gay, M.A.; Muñiz-Montes, J.R.; Gómez Rodríguez-Bethencourt, M.A.; González-Díaz, A.; Díaz-González, F.; Ferraz-Amaro, I. Relationship of abdominal adiposity and body composition with endothelial dysfunction in patients with rheumatoid arthritis. *Clin. Exp. Rheumatol.* **2015**, *33*, 516–523.
29. Roubenoff, R.; Roubenoff, R.A.; Cannon, J.G.; Kehayias, J.J.; Zhuang, H.; Dawson-Hughes, B.; Dinarello, C.A.; Rosenberg, I.H. Rheumatoid cachexia: Cytokine-driven hypermetabolism accompanying reduced body cell mass in chronic inflammation. *J. Clin. Invest.* **1994**, *93*, 2379–2386, doi:10.1172/JCI117244.
30. Little, R.D.; Prieto-Potin, I.; Pérez-Baos, S.; Villalvilla, A.; Gratal, P.; Cicuttini, F.; Largo, R.; Herrero-Beaumont, G. Compensatory anabolic signaling in the sarcopenia of experimental chronic arthritis. *Sci. Rep.* **2017**, *7*, 6311, doi:10.1038/s41598-017-06581-6.
31. Visser, M.; Pahor, M.; Taaffe, D.R.; Goodpaster, B.H.; Simonsick, E.M.; Newman, A.B.; Nevitt, M.; Harris, T.B. Relationship of interleukin-6 and tumor necrosis factor-alpha with muscle mass and muscle strength in elderly men and women: the Health ABC Study. *J. Gerontol. A. Biol. Sci. Med. Sci.* **2002**, *57*, M326-32, doi:10.1093/gerona/57.5.m326.
32. de Oliveira Nunes Teixeira, V.; Filippin, L.I.; Viacava, P.R.; de Oliveira, P.G.; Xavier, R.M. Muscle wasting in collagen-induced arthritis and disuse atrophy. *Exp. Biol. Med.* **2013**, *238*, 1421–1430, doi:10.1177/1535370213505961.
33. Castillero, E.; Martín, A.I.; López-Menduiña, M.; Granado, M.; Villanúa, M.Á.; López-Calderón, A. IGF-I system, atrogenes and myogenic regulatory factors in arthritis induced muscle wasting. *Mol. Cell. Endocrinol.* **2009**, *309*, 8–16, doi:10.1016/j.mce.2009.05.017.
34. El Maghraoui, A.; Ebo'O, F.B.; Sadni, S.; Majjad, A.; Hamza, T.; Mounach, A. Is there a relation between pre-sarcopenia, sarcopenia, cachexia and osteoporosis in patients with ankylosing spondylitis? *BMC Musculoskelet. Disord.* **2016**, *17*, 268, doi:10.1186/s12891-016-1155-z.
35. Krajewska-Wlodarczyk, M.; Owczarczyk-Saczonek, A.; Placek, W. Changes in body composition and bone mineral density in postmenopausal women with psoriatic arthritis. *Reumatologia* **2017**, *55*, 215–221, doi:10.5114/reum.2017.71627.
36. Janssen, I.; Heymsfield, S.B.; Ross, R. Low relative skeletal muscle mass (sarcopenia) in older persons is associated with functional impairment and physical disability. *J. Am. Geriatr. Soc.* **2002**, *50*, 889–896, doi:10.1046/j.1532-5415.2002.50216.x.
37. Aguiar, R.; Sequeira, J.; Meirinhos, T.; Ambrósio, C.; Barcelos, A. SARCOSPA-sarcopenia in spondyloarthritis patients. *Acta Reumatol. Port.* **2014**, *2014*, 322–326.
38. Caimmi, C.; Caramaschi, P.; Venturini, A.; Bertoldo, E.; Vantaggiato, E.; Viapiana, O.; Ferrari, M.; Lippi, G.; Frulloni, L.; Rossini, M. Malnutrition and sarcopenia in a large cohort of patients with systemic sclerosis. *Clin. Rheumatol.* **2018**, *37*, 987–997, doi:10.1007/s10067-017-3932-y.
39. Siegert, E.; March, C.; Otten, L.; Makowka, A.; Preis, E.; Buttgereit, F.; Riemekasten, G.; Müller-Werdan, U.; Norman, K. Prevalence of sarcopenia in systemic sclerosis: Assessing body composition and functional disability in patients with systemic sclerosis. *Nutrition* **2018**, *55–56*, 51–55, doi:10.1016/j.nut.2018.03.046.
40. Corallo, C.; Fioravanti, A.; Tenti, S.; Pecetti, G.; Nuti, R.; Giordano, N. Sarcopenia in systemic sclerosis: the impact of nutritional, clinical, and laboratory features. *Rheumatol. Int.* **2019**, *39*, 1767–1775, doi:10.1007/s00296-019-04401-w.
41. Pacini, G.; Paolino, S.; C.; Trombetta, A.; Goegan, F.; Pizzorni, C.; Alessandri, E.; Patanè, M.; Gotelli, E.; Ferrari, G.; Cattelan, F.; et al. Lower urinary tract symptoms in systemic sclerosis: a detailed investigation. *Rheumatology (Oxford).* **2020**, *59*, 1315–1324, doi:10.1093/rheumatology/kez438.
42. Justo, A.C.; Guimarães, F.S.; Ferreira, A.S.; Soares, M.S.; Bunn, P.S.; Lopes, A.J. Muscle function in women with systemic sclerosis: Association with fatigue and general physical function. *Clin. Biomech.* **2017**, *47*, 33–39, doi:10.1016/j.clinbiomech.2017.05.011.
43. Marighela, T.F.; Genaro, P.D.S.; Pinheiro, M.M.; Szejnfeld, V.L.; Kayser, C. Risk factors for body composition abnormalities in systemic sclerosis. In Proceedings of the Clinical Rheumatology; *Clin Rheumatol*, **2013**; *32*, 1037–1044, doi:10.1007/s10067-013-2235-1.

44. Doerfler, B.; Allen, T.S.; Southwood, C.; Brenner, D.; Hirano, I.; Sheean, P. Medical Nutrition Therapy for Patients with Advanced Systemic Sclerosis (MNT PASS): A Pilot Intervention Study. *J. Parenter. Enter. Nutr.* **2017**, *41*, 678–684, doi:10.1177/0148607115597883.
45. Adams, D.W.; Gurwara, S.; Silver, H.J.; Horst, S.N.; Beaulieu, D.B.; Schwartz, D.A.; Seidner, D.L. Sarcopenia Is Common in Overweight Patients with Inflammatory Bowel Disease and May Predict Need for Surgery. *Inflamm. Bowel Dis.* **2017**, *23*, 1182–1186, doi:10.1097/MIB.0000000000001128.
46. Prado, C.M.; Lieffers, J.R.; McCargar, L.J.; Reiman, T.; Sawyer, M.B.; Martin, L.; Baracos, V.E. Prevalence and clinical implications of sarcopenic obesity in patients with solid tumours of the respiratory and gastrointestinal tracts: a population-based study. *Lancet Oncol.* **2008**, *9*, 629–635, doi:10.1016/S1470-2045(08)70153-0.
47. Bryant, R.V.; Schultz, C.G.; Ooi, S.; Goess, C.; Costello, S.P.; Vincent, A.D.; Schoeman, S.N.; Lim, A.; Bartholomeusz, F.D.; Travis, S.P.L.; et al. Obesity in inflammatory bowel disease: Gains in adiposity despite high prevalence of Myopenia and Osteopenia. *Nutrients* **2018**, *10*, 1192, doi:10.3390/nu10091192.
48. Pedersen, M.; Cromwell, J.; Nau, P. Sarcopenia is a Predictor of Surgical Morbidity in Inflammatory Bowel Disease. *Inflamm. Bowel Dis.* **2017**, *23*, 1867–1872, doi:10.1097/MIB.0000000000001166.
49. Werkstetter, K.J.; Ullrich, J.; Schatz, S.B.; Prell, C.; Koletzko, B.; Koletzko, S. Lean body mass, physical activity and quality of life in paediatric patients with inflammatory bowel disease and in healthy controls. *J. Crohns. Colitis* **2012**, *6*, 665–673, doi:10.1016/j.crohns.2011.11.017.
50. Mager, D.R.; Carroll, M.W.; Wine, E.; Siminoski, K.; MacDonald, K.; Kluthe, C.L.; Medvedev, P.; Chen, M.; Wu, J.; Turner, J.M.; et al. Vitamin D status and risk for sarcopenia in youth with inflammatory bowel diseases. *Eur. J. Clin. Nutr.* **2018**, *72*, 623–626, doi:10.1038/s41430-018-0105-2.
51. Webber, C.E.; Barr, R.D. Age and gender-dependent values of skeletal muscle mass in healthy children and adolescents. *J. Cachexia. Sarcopenia Muscle* **2012**, *3*, 25–29, doi:10.1007/s13539-011-0042-6.
52. Zhang, T.; Cao, L.; Cao, T.; Yang, J.; Gong, J.; Zhu, W.; Li, N.; Li, J. Prevalence of Sarcopenia and Its Impact on Postoperative Outcome in Patients with Crohn's Disease Undergoing Bowel Resection. *J. Parenter. Enter. Nutr.* **2017**, *41*, 592–600, doi:10.1177/0148607115612054.
53. Dindo, D.; Demartines, N.; Clavien, P.A. Classification of surgical complications: A new proposal with evaluation in a cohort of 6336 patients and results of a survey. *Ann. Surg.* **2004**, *240*, 205–213.
54. Fearon, K.; Strasser, F.; Anker, S.D.; Bosaeus, I.; Bruera, E.; Fainsinger, R.L.; Jatoi, A.; Loprinzi, C.; MacDonald, N.; Mantovani, G.; et al. Definition and classification of cancer cachexia: An international consensus. *Lancet Oncol.* **2011**, *12*, 489–495, doi: 10.1016/S1470-2045(10)70218-7.
55. Bamba, S.; Sasaki, M.; Takaoka, A.; Takahashi, K.; Imaeda, H.; Nishida, A.; Inatomi, O.; Sugimoto, M.; Andoh, A. Sarcopenia is a predictive factor for intestinal resection in admitted patients with Crohn's disease. *PLoS One* **2017**, *12*, e0180036, doi:10.1371/journal.pone.0180036.
56. Nishikawa, H.; Shiraki, M.; Hiramatsu, A.; Moriya, K.; Hino, K.; Nishiguchi, S. Japan Society of Hepatology guidelines for sarcopenia in liver disease (1st edition): Recommendation from the working group for creation of sarcopenia assessment criteria. *Hepatol. Res.* **2016**, *46*, 951–963, doi:10.1111/hepr.12774.
57. Thiberge, C.; Charpentier, C.; Gillibert, A.; Modzelewski, R.; Dacher, J.-N.; Savoye, G.; Savoye-Collet, C. Lower Subcutaneous or Visceral Adiposity Assessed by Abdominal Computed Tomography Could Predict Adverse Outcome in Patients With Crohn's Disease. *J. Crohns. Colitis* **2018**, *12*, 1429–1437, doi:10.1093/ecco-jcc/jjy124.
58. Mourtzakis, M.; Prado, C.M.M.; Lieffers, J.R.; Reiman, T.; McCargar, L.J.; Baracos, V.E. A practical and precise approach to quantification of body composition in cancer patients using computed tomography images acquired during routine care. *Appl. Physiol. Nutr. Metab.* **2008**, *33*, 997–1006, doi:10.1139/H08-075.
59. Lee, C.H.; Yoon, H.; Oh, D.J.; Lee, J.M.; Choi, Y.J.; Shin, C.M.; Park, Y.S.; Kim, N.; Lee, D.H.; Kim, J.S. The prevalence of sarcopenia and its effect on prognosis in patients with Crohn's disease. *Intest. Res.* **2020**, *18*, 79–84, doi:10.5217/ir.2019.00107.
60. Kim, Y.-S.; Lee, Y.; Chung, Y.-S.; Lee, D.-J.; Joo, N.-S.; Hong, D.; Song, G. eun; Kim, H.-J.; Choi, Y.J.; Kim, K.-M. Prevalence of sarcopenia and sarcopenic obesity in the Korean population based on the Fourth Korean National Health and Nutritional Examination Surveys. *J. Gerontol. A. Biol. Sci. Med. Sci.* **2012**, *67*, 1107–1113, doi:10.1093/gerona/gls071.

61. Cravo, M.L.; Velho, S.; Torres, J.; Costa Santos, M.P.; Palmela, C.; Cruz, R.; Strecht, J.; Maio, R.; Baracos, V. Lower skeletal muscle attenuation and high visceral fat index are associated with complicated disease in patients with Crohn's disease: An exploratory study. *Clin. Nutr. ESPEN* **2017**, *21*, 79–85, doi:10.1016/j.clnesp.2017.04.005.
62. Martin, L.; Birdsell, L.; MacDonald, N.; Reiman, T.; Clandinin, M.T.; McCargar, L.J.; Murphy, R.; Ghosh, S.; Sawyer, M.B.; Baracos, V.E. Cancer cachexia in the age of obesity: Skeletal muscle depletion is a powerful prognostic factor, independent of body mass index. *J. Clin. Oncol.* **2013**, *31*, 1539–1547, doi:10.1200/JCO.2012.45.2722.
63. Carvalho, D.; Viana, C.; Marques, I.; Costa, C.; Martins, S.F. Sarcopenia is associated with Postoperative Outcome in Patients with Crohn's Disease Undergoing Bowel Resection. *Gastrointest. Disord.* **2019**, *1*, 201–209, doi:10.3390/gidisord1010015.
64. Ding, N.S.; Malietzis, G.; Lung, P.F.C.; Penez, L.; Yip, W.M.; Gabe, S.; Jenkins, J.T.; Hart, A. The body composition profile is associated with response to anti-TNF therapy in Crohn's disease and may offer an alternative dosing paradigm. *Aliment. Pharmacol. Ther.* **2017**, *46*, 883–891, doi:10.1111/apt.14293.
65. Subramaniam, K.; Fallon, K.; Ruut, T.; Lane, D.; McKay, R.; Shadbolt, B.; Ang, S.; Cook, M.; Platten, J.; Pavli, P.; et al. Infliximab reverses inflammatory muscle wasting (sarcopenia) in Crohn's disease. *Aliment. Pharmacol. Ther.* **2015**, *41*, 419–428, doi:10.1111/apt.13058.
66. Zhang, T.; Ding, C.; Xie, T.; Yang, J.; Dai, X.; Lv, T.; Li, Y.; Gu, L.; Wei, Y.; Gong, J.; et al. Skeletal muscle depletion correlates with disease activity in ulcerative colitis and is reversed after colectomy. *Clin. Nutr.* **2017**, *36*, 1586–1592, doi:10.1016/j.clnu.2016.10.004.
67. Cushing, K.C.; Kordbacheh, H.; Gee, M.S.; Kambadakone, A.; Ananthakrishnan, A.N. Sarcopenia is a novel predictor of the need for rescue therapy in hospitalized ulcerative colitis patients. *J. Crohn's Colitis* **2018**, *12*, 1036–1041, doi:10.1093/ecco-jcc/jjy064.
68. Fujikawa, H.; Araki, T.; Okita, Y.; Kondo, S.; Kawamura, M.; Hiro, J.; Toiyama, Y.; Kobayashi, M.; Tanaka, K.; Inoue, Y.; et al. Impact of sarcopenia on surgical site infection after restorative proctocolectomy for ulcerative colitis. *Surg. Today* **2017**, *47*, 92–98, doi:10.1007/s00595-016-1357-x.
